# Lessons From Extreme Altitudes

**DOI:** 10.3389/fphys.2019.00703

**Published:** 2019-06-07

**Authors:** John B. West

**Affiliations:** Department of Medicine, University of California, San Diego, La Jolla, CA, United States

**Keywords:** AMREE, altitude, silver hut expedition, Everest, hypoxia

## Introduction

I have been fortunate to participate in two major physiological research expeditions to extreme altitude. The first was the Silver Hut expedition in 1960–1961 during which a group of physiologists spent several months at the very high altitude of 5,800 m (19,000 ft), and we measured the physiological changes that occurred over this long period. Additional studies were made up to 7,830 m. (25,700 ft). The overall purpose was to determine the mechanisms by which people who normally live near sea level respond to severe hypoxia over an extended period.

The second expedition was the American Medical Research Expedition to Everest which occurred in 1981. Here the physiological aim was very different. The objective was to obtain the first human physiological data on the summit of Mount Everest (8,848 m, 29,028 ft) in order to clarify how people who normally live at sea level can survive the extreme hypoxia of the highest point in the world.

## Silver Hut Expedition

This was the brain-child of Sir Edmund Hillary who, together with Tenzing Norgay, was the first person to reach the summit of Mt Everest seven years before. Hillary had collaborated with Griffith Pugh, a high-altitude physiologist, on this first ascent of Everest, and both men were intensely interested in the acclimatization process that enables people from near sea level to climb to very high altitudes.

My particular interest was the diffusing capacity of the lung. It had been suggested by Barcroft (1925) that exercise at high altitude would result in arterial hypoxemia because of diffusion limitation across the blood-gas barrier. To test this, we arranged for the expedition members to work at their maximal capacity on a cycle ergometer, and we measured the arterial oxygen saturation using a newly available ear oximeter (West et al., 1962). We found that there was in fact a marked fall in the arterial oxygen saturation in spite of the increase in the alveolar PO_2_ as the work level was raised. This was strong evidence of diffusion limitation under these conditions of severe hypoxia. Duplicate measurements on myself showed an oxygen saturation of only 33% at maximum exercise which reflected very severe hypoxemia.

We also measured the diffusing capacity for carbon monoxide over the course of the expedition and showed that this hardly changed (West, 1962). The small increase could be explained by the polycythemia which developed. The conclusion was that the characteristics of the blood gas barrier were not altered by prolonged exposure to severe hypoxia. This was the first clear demonstration of diffusion limitation of oxygen transfer by the lung during severe exercise at very high altitude.

Later in the expedition, measurements were made of the maximal oxygen uptake during exercise at the extremely high altitude of 7,440 m (24,400 ft) (Pugh et al., 1964). Extrapolation of these data to the altitude of the Everest summit suggested that it would be impossible to reach the summit without supplementary oxygen. Blood studies showed marked polycythemia in the subjects living at an altitude of 5,800 m. The mean hemoglobin concentrations and hematocrits were 19.6 g/dl and 55.8%, respectively. There was evidence that the initial increase in hematocrit was mainly caused by loss of plasma volume, but later there was a large increase in red cell mass. The electrocardiogram of people living at 5,800 m showed marked right ventricular hypertrophy, and in some tracings there was inversion of the T waves in the chest leads, presumably indicating severe myocardial hypoxia (Milledge, 1963). Measurements of neuropsychometric function were made using card sorting, and it was found that the rate of sorting was reduced but that with increased concentration the subjects could sort without errors. There was a severe, relentless weight loss in all the members of the expedition while living at 5,800 m with the rate being between 0.5 and 1.5 Kg/week. The general impression was that it would not be possible for people to live indefinitely at this very high altitude (Pugh, 1962).

## American Medical Research Expedition to Everest

As noted above, the aim of this expedition was to clarify how humans can tolerate the hypoxia of the highest altitude in the world. Remarkably, a few months before the expedition took place, two European climbers reached the summit of Mount Everest for the first time without using supplementary oxygen. This feat astonished many physiologists, and raised many questions about how it could be done.

The research program was very extensive, and only a brief summary can be given here. Measurements were made at the base camp, altitude 5,400 m (17,700 ft), and the advanced base camp, altitude 6,300 m (20,700 ft), and at the highest camp 8,050 m (26,400 ft). We also hoped to get some measurements on the Everest summit although this was very ambitious. In fact, when we looked back on the six expeditions prior to our own, not one of them reached the summit. If the weather is bad, forget it, and a critical factor is whether sufficient members of the expedition remain fit enough in spite of the extreme altitude.

At the base camp, we measured the ventilatory response to hypoxia, that is the extent to which breathing increases when the subject is exposed to a low inspired oxygen mixture. The results were striking. It turned out that the climber who got to the summit first had the highest response, the one who got to the summit second had the next highest response, and the climber who got to the summit third had the third highest response (Schoene et al., 1984). This must be partly by chance but it certainly suggested that there was a link between the extent to which climbers increase their ventilation, and their tolerance to extreme altitude. The reason for this will become clearer below.

A large number of studies were carried out at the advanced base camp but only one, a neuropsychometric study, will be summarized here. It is well-known that the brain and central nervous system are very sensitive to hypoxia. For example, if somebody falls into a swimming pool and is rescued 5 or 10 min later, he may be successfully revived but the central nervous system never completely recovers. It was therefore not surprising that we could show changes in measurements such as short-term memory and manipulative skill (as determined from a finger tapping test) at the very high altitudes. This was not unexpected. However, when the expedition returned to near sea level, it was found that two of the measurements remained abnormal. These were the short-term memory and the finger tapping test (Townes et al., 1984). It was therefore clear that anybody returning from these extreme altitudes is likely to have some residual impairment of the central nervous system. We were one of the first groups to show this but it has been confirmed many times since.

Some of the most interesting findings were those from the summit. We had designed and built a special device that enabled the climber to collect the last expired gas after a maximal expiration, that is an alveolar gas sample. Over 34 samples including four from the summit were brought back to UC San Diego in gas tight cans. When the alveolar P_CO2_ was plotted against the barometric pressure which fell as altitude increased, it was found that the P_CO2_ on the summit was 7–8 mm Hg. This was an almost unbelievably low value since the sea level value is about 40 mm Hg. This extremely low value emphasizes the enormous increase in alveolar ventilation that is necessary at these extreme altitudes (West et al., 1983).

**Figure 1:**
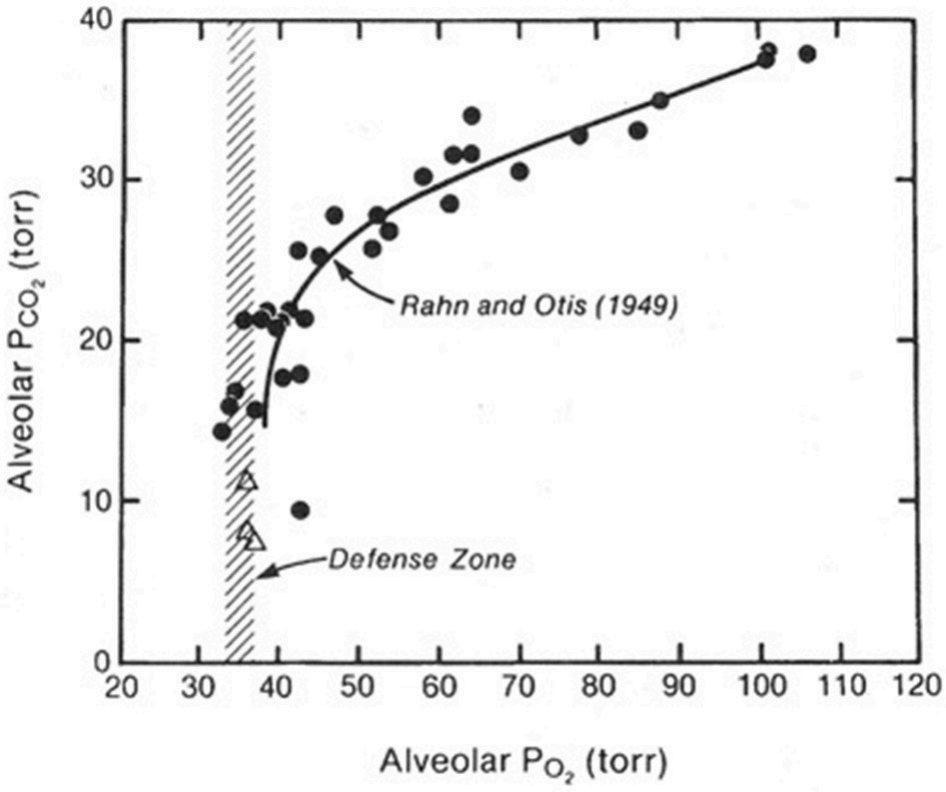
Values of the PO_2_ and P_CO2_ in the alveolar gas of climbers as they ascend from sea level (top right) to the Everest summit (bottom left). The PO_2_ falls because of the reduction in barometric pressure. The P_CO2_ falls because of the increasing alveolar ventilation. Above an altitude corresponding to a P_CO2_ of about 20 mm Hg (about 7000 m), there is no further fall in the PO_2_. In other words, this is defended at about 35 mmHg. This can only be done if the P_CO2_ is continually reduced further by extreme hyperventilation as the altitude rises. Modified from Rahn and Otis (1949) and West et al. (1983).

When both the alveolar PO_2_ and P_CO2_ were plotted against barometric pressure, and interesting picture emerged (Figure 1). Both the PO_2_ and the P_CO2_ fell as the altitude increased. The fall in PO_2_ occurred because of the reduction in the air around the climber as a result of the reduction in barometric pressure. The fall in the P_CO2_ was caused only by the climber's hyperventilation. It transpired that when the altitude exceeded about 7000 m, there was no further fall in the alveolar PO_2_. The figure shows that this is defended at a level of about 35 mm Hg. In other words, in order to survive at these enormous altitudes, you need to mount an alveolar ventilation that will drive the P_CO2_ down to around 8 mmHg and thus preserve the alveolar PO_2_ at the very low but viable level of about 35 mm Hg. This explains why in the measurements of the ventilatory response to hypoxia referred to earlier, there was a correlation between the magnitude of the response and the tolerance of the climber to extreme altitude. If you are not able to mount a degree of hyperventilation that is sufficient to drive the alveolar P_CO2_ down to about 7–8 mm Hg you cannot maintain a viable level of PO_2_ in the alveolar gas. Thus, extreme hyperventilation is one of the most important features of the physiological response to extremely high altitude.

The extremely low alveolar P_CO2_ prompts the question of what happened to the arterial pH. It is reasonable to assume that the arterial and alveolar P_CO2_ are the same. Fortunately, two of the climbers took venous blood from each other on the morning after their successful climb to the summit, and the base excess values could therefore be measured. When these values were entered into the Henderson-Hasselbalch equation, the resulting arterial pH was between 7.7 and 7.8. This is an extreme degree of respiratory alkalosis.

An interesting question is why the kidney did not eliminate bicarbonate to develop a metabolic compensation for this extreme alkalosis and thus move the pH closer to normal. This is the usual response if a respiratory alkalosis is generated, for example, by hyperventilation as sometimes occurs during hysteria. The reason for the absence of metabolic compensation is not completely understood, but a possibility is that these climbers were probably severely volume-depleted. This is a common feature of going to high altitude and, for example, the climbers who were staying at the advanced base camp at 6300 m showed evidence of chronic volume depletion. One responsible factor at extreme altitude is presumably the enormous hyperventilation, but thirst is impaired as well.

The physiological consequences of the severe alkalosis are interesting. Other studies have shown that an increased oxygen affinity of hemoglobin increases survival in a hypoxic environment. Many years ago, it was shown that mammals such as the vicuña and llama in the South American Andes have an increased oxygen affinity (that is they had left -shifted oxygen dissociation curve) compared with mammals living at sea level (Hall, 1937). Thus, climbers at very high altitude have the same response.

It is also true that if you look generally throughout the animal kingdom at organisms that live in an hypoxic environment, many of them have developed an increased oxygen affinity of the hemoglobin. One of the best examples is the human fetus that, because of a difference in the amino acid sequence of the hemoglobin, has a marked increase in oxygen affinity with P_50_ of about 19 mm Hg compared with that of about 27 for an adult. The human fetus has severe hypoxemia by adult standards with a PO_2_ in the descending aorta of about 22 mm Hg which is even lower than that of a climber on the Everest summit. It is fascinating indeed that the successful climber has the advantage of an increased oxygen affinity of the hemoglobin. This assists in the loading of oxygen in the pulmonary capillary. It could be argued that it also interferes with the unloading of oxygen in the periphery of the body, but studies have shown that the advantage of loading in the lung is greater than the disadvantage in unloading in the peripheral tissues.

An interesting question is what is the maximal oxygen consumption of a climber on the summit. As indicated earlier, previous measurements made at extreme altitude during the Silver Hut expedition suggested that all the oxygen available on the summit would be required for the basal oxygen update, that is to keep the heart pumping and the brain active. It was impossible to put a cycle ergometer on the summit. However, we took climbers who were very well acclimatized and had them pedal maximally at an altitude of 6300 m while breathing 14% oxygen. This gave them the same inspired PO_2_ as on the summit. The oxygen uptake level under these conditions was about 1 L/min which is a miserably low maximal oxygen consumption, and is equivalent to somebody walking slowly on the level. However, it is apparently just sufficient to enable a climber to reach the summit.

## Author Contributions

JW drafted the manuscript, read, and approved the submitted version.

### Conflict of Interest Statement

The author declares that the research was conducted in the absence of any commercial or financial relationships that could be construed as a potential conflict of interest.
